# History of the Epidemic Yellow Fever at New Orleans, La., in 1853

**Published:** 1854-05

**Authors:** 


Art. IV.—*History of the Epidemic Yellow Fever at New Orleans, La., in* 1853, by E. D. Fenner, M. D.

*Tableau of the Yellow Fever of* 1853, *with Topographical, Chronological and Historical Sketches of the Epidemics of New Orleans, since their origin in* 1796, *illustrative of the Quarantine Question*, by Bennet Dowler, M. D.

We resume in our present number the analysis of the histories of Drs. Fenner and Dowler, and shall make such extracts from both, without much comment, as may suffice to illustrate the several remaining topics of which they treat.

Both papers contain very full statistics of the epidemic; these have been collected with great care and are as accurate as statistics usually are, under more favorable circumstances. There is some discrepancy in the figures of the two, due partly to the difference in the dates, but this does not materially alter the result. We shall transfer the statistics of Dr. Fenner showing the number of cases that occurred and the mortality, in the five months from the 1st of June to the 1st of November.

"*Mortality of the Epidemic.*"

| Months. | Total Deaths. | Yellow Fever. | Other Diseases. | Not Stated. |
|---|---|---|---|---|
| June, | 665 | 49 | 581 | 45 |
| July, | 2077 | 1407 | 559 | 112 |
| August, | 6235 | 5189 | 689 | 357 |
| September, | 1681 | 1070 | 489 | 113 |
| October, | 676 | 265 | 438 | 73 |
| | 11,335 | 7870 | 2765 | 700 |

"This table presents the deaths from Yellow Fever, as shown on the *burial certificates*. It is generally admitted that *six-sevenths*

of the certificates in which the cause of death was *not stated* during the epidemic should be added to the yellow fever list, which, being 600, would make that list amount to 8,470. (The table of Dr. DOWLER makes the number 8,400.) Taking this as correct, and having *some certain data* to go by, we may now make an *approximative* calculation of the whole number of cases of yellow fever that occurred in the city during the five months above specified, and also the general rate of mortality for the whole.

| | Cases. | Deaths. | Loss per cent. |
|---|---|---|---|
| Charity Hospital, | 3052 | 1765 | 46 |
| Howard Association, | 10500 | 3150 | 30 |
| Maison de Santé, | 338 | 97 | 28 |
| City Workhouse, 1st dist. | 89 | 13 | 15 |
| Touro Infirmary, | 523 | 213 | 40 |
| Boy's House Refuge, | 21 | 6 | 28 |
| Girl's House Refuge, | 21 | 1 | 5 |
| City Prison, 2d dist. | 30 | 5 | 16 |
| Lunatic Asylum, | 8 | 0 | 0 |
| Board Health Infirmary, No. 1, | 343 | 155 | 45 |
| " " " No. 2, | 423 | 207 | 48 |
| Totals, | 15,858 | 5,613 | 311 |

"We thus have 15,858 cases, with a loss of 5,613, which is at the rate of 35½ per cent., inasmuch as all these deaths were certified by physicians; when deducted from 7,870, the total of the same class, it leaves a balance of 2,257 deaths, to be distributed amongst other hospitals and infirmaries, asylums and private practice. If we consider these 2,257 deaths to be 20 per cent. of the whole number of cases treated in the last mentioned quarters, it would show 11,285 cases, which added to 15,858, the number of cases above ascertained, would give a probable grand total of 27,143 cases treated in the city during the time specified.

"Estimating the probable population of the city at 100,000, during the epidemic, it thus appears that upwards of *one-fourth* of them had the fever; and of those who were attacked, within a fraction of 29 per cent. died, being nearly 8 per cent, of the estimated population. Of course these results would have to be different if we either raise the amount estimated for the population, or add to the list of *certified deaths* by yellow fever six-sevenths of those in which the cause of death was *not stated* during the prevalence of the epidemic. Every other city or town visited by this epidemic was at least *decimated*, and some of them more."

"The great epidemic of 1847 only caused about 3000 deaths in this city and Lafayette, which were then separate, but are now united."

During the prevalence of yellow fever, which by its malignity and unprecedented mortality almost exclusively absorbed public attention, other forms of fever, such as typhus, typhoid, remittent, intermittent, congestive and scarlet, nevertheless existed, and contributed to swell the number of deaths; the *mortuary* reports of the Board of Health, during the months of June, July, August, and September, show that "in the month of June the deaths from all the fevers specified amount to 143, of which yellow fever was 46. In July, all fevers 1482, yellow fever 1380. In August, all fevers 4962, yellow fever 4791. In September, all fevers 779, yellow fever 722. The total number of deaths from *all fevers* during these four months, as published, was 7472; of which 6945 died of yellow fever, and 527 died of other types."

"From these *mortuary* statistics it will be seen that during the reign of the *great epidemic*, which slew its thousands, people likewise died of *all sorts of fevers.* The statistics of the *living*, which I shall now present from the Charity Hospital, also show a similar variety of fevers prevailing together, though in somewhat different proportions." We omit the table of patients received into the Charity Hospital during the four months specified, but learn from it that there were admitted of *all diseases* 6037, of which 4370 were for *fever of all kinds*, 3052 were yellow fever, 942 were intermittent, and a varying proportion of the other types of fever, for the several months.

Dr. Dowler presents some interesting details touching the prevalence of other diseases and the rates of mortality from them during the existence of the epidemic. He says, "as the epidemic increased the mortality from other causes was little affected, particularly after the month of July. For five months, ending June 25th, the average weekly mortality from yellow fever was about 4, from other diseases 116; the next five weeks gave an average for yellow fever of 280 per week, other diseases 160. The next four weeks ending August 28, gave a yellow fever average mortality of 1,211 per week, or by weeks respectively, 967, 1,288, 1,346, 1,243, aggregate 4,844; while for the same time, the average from other causes was 157, not including an average weekly mortality of 71, reported as deaths from causes unknown, and generally supposed to have been deaths from yellow fever. During the next four weeks ending September 28th, the average weekly deaths from yellow fever was nearly 200, while for the same time the average from other known causes gave 65, and deaths from unknown causes a fraction over 16."

"From the last week in May, when the weekly mortality was for yellow fever 1, and for all other diseases 139, the non-yellow fever mortality vibrated but little until after the week ending the 23d of July, having ranged from its maximum 158, for the week ending 25th of July to its minimum 129, for the week ending July 9th, but for the week ending July 23d it rose to 188; the next week it reached its maximum for the season, that is 192, at which time the epidemic was rapidly increasing, 1,409 having died, 1,121 in the two preceding, and 1,325 in the three preceding weeks. About the 9th of July, the epidemic, as such, began, so that the ratio of mortality from causes other than yellow fever, was not much disturbed during the greatest fury of the epidemic until the week ending September 4th, after which it declined to 102; but for the next week it rose to 120, nine less than on the 9th of July, when the epidemic appeared, and 19 less than the week ending May 28th when the first death from yellow fever was reported. In the week ending the 21st of August, which gave the maximum of deaths from yellow fever, that is 1,346, the mortality from other diseases was but 152, exactly the same as for the week ending July 2nd, when, as yet, all the deaths from yellow fever beginning with May, were but 47. So that the ratio of mortality, independent of the yellow fever element during its unparalleled prolongation was but little changed except during the last two precursory weeks of July. Thus the epidemic caused but a slight oscillation among other maladies, while its pestilential waves rolled over the devoted city."

"If the epidemic be considered in a larger view, by months, it will be seen that its collateral influence is scarcely manifest. Mortality from causes other than yellow fever, for June, 581; for July, 559; for yellow fever for June, 40; for July, 1,406, not including 45 for the former month, and 112 for the latter, under the head of unknown or *not stated*."

"The greatest number of deaths in any one day, 283, of which 239 were from yellow fever, occurred on the 22d of August. The greatest number of deaths from the fever, in any one month, was in August, amounting to 5,189, or by adding the unknown, 5,242; by adding all the deaths, 6,235; an average exceeding 201 per day; about 9 every hour; one every six or seven minutes! for a whole month!!"

Dr. FENNER gives several tables showing the number of cases of the various forms of fever and their mortality, and reiterates the opinion previously stated as to the probable identity of cause. "It is quite common to see these various types of fever interchange or run into each other, just as is seen in the country where the intermittent and bilious remittent are supposed to originate from the same remote cause."

Dr. Dowler furnishes some interesting facts in regard to the ratio of mortality of the sexes and among children. Upon these points Dr. Fenner gives no information in detail. Dr. Dowler says, "according to the report of the Howard Association, published late in December, the Society had under its care during the epidemic of 1853, no less than 11,088 yellow fever patients—5,203 males, 5,885 females—of whom 2,942 died and 8,146 were cured. The predominance of female patients in the above enumeration is remarkable, inasmuch as that sex is the least susceptible to the yellow fever, and contribute to the mortality from this disease in a ratio greatly inferior to males. The most probable explanation is this—females preferred the Howard Hospitals to the Charity Hospital, and the City Hospitals established by the Board of Health."

"In order to ascertain approximatively the proportion between the mortality of the sexes, I selected the first day of August, counting all the interments from fever, as distributed among the letters of the alphabet, and among the following cemeteries, in Mr. Maginnis' List of Interments:"

| | Males. | Females. |
|---|---|---|
| Cypress Grove, No. 2, | 3 | 2 |
| Protestant, | 1 | 1 |
| Charity Hospital, | 21 | 9 |
| Lafayette, | 24 | 11 |
| St. Patrick's, | 15 | 10 |
| St. Vincent, | 13 | 8 |
| Total, | 82 | 41 |

"Hence it appears that the mortality of females, is, for 1853, exactly half as great as that of males. This high ratio of female mortality is, however, one of the most extraordinary features of the late epidemic. Of 1,450 who died of yellow fever in August, September and October, 1841, but 220 were females, or nearly one in seven. The ratio of mortality among children will probably be found enormously high from fever in 1853, compared with preceding years. This will appear obvious by Mr. Maginnis' list, compared with the following extensive analysis of the epidemic of 1841; thus—I made thirty-three series, each consisting of thirty persons, I then took the youngest one in each series, (among these 990 dead) which gave these ages: 15, 17, 17, 2, 5, 20, 19, 16, 20, 17, 15, 17, 18, 19, 8, 2, 7, 18, 18, 19, 8, 6, 8, 2, 15, 3, 18, 14, 2, 18, 3, 5, 19. Scarcely an infant in the whole series!"

"In order to test approximatively, the ratio of infantile deaths from fever, I counted the ages of all fever victims who were interred in the following cemeteries, on the 10th of August, namely, Cypress Grove, No. 1 and No. 2, and St. Patrick's, amounting to eighty-nine known ages, and two called 'infants,' (say ninety-one) among which were two aged 2; one aged 3; one 4; which with the two infants, make six out of ninety-one—a result which could not have been anticipated from the history of anterior epidemics, as the very young and very old, as well as women and negroes, had always suffered less than other classes."

The observation of Dr. FENNER accords with the statistics of Dr. DOWLER in regard to the prevalence of the disease among children in 1853; he remarks, "one of the most extraordinary features of this epidemic is presented in the fact that the natives of the city, *both white and colored*, have suffered severely, and many of them died of it. This is generally admitted, and beyond dispute. Children who were born since '47 have suffered most; but many born previous to that time likewise suffered, and some of them died of black vomit. It may be stated generally, that all children, and young persons in the city, who had never had yellow fever previously, were attacked this year. Of course there are some exceptions to this, as there are to every general rule."

Although the mortality was greater among negroes during the epidemic of 1853 than was ever before known, it was not sufficient to invalidate the opinion based upon past experience as to the immunity enjoyed by the African race. Upon this point, Dr. FENNER speaks in general terms to the following effect: "This epidemic affected unacclimated negroes, or those who had never had yellow fever before, equally as generally as it did the white population, though not so severely. This is equally true of the mixed races generally. It is a well established fact that there is something in the negro constitution which affords him protection against the worst effects of yellow fever; but what it is I am unable to say. During an epidemic he will take the fever almost, if not fully as readily as the white, but it will be altogether milder and less dangerous in its tendency. In short, it will correspond more exactly with the bilious remittent fever that prevails in the country, and requires precisely the same treatment. And yet this type of fever in the city negro must be produced by the very same cause that gives rise to malignant yellow fever in the white race. Occasionally we see the hemorrhagic diathesis of yellow fever displayed in the negro, but it is by no means common. The least mixture of the *white race* with the *black* seems to increase the liability of the latter to the dangers of yellow fever; and the danger is in proportion to the amount of white blood in the mixture. Very few negroes ever die of yellow fever in this city; but I learn that a considerable number have been lost on the plantations this year. The cause of this may be readily imagined when it is recollected that yellow fever never prevailed on the plantations before, and of course most of the physicians were not familiar with its treatment."

"I consider the danger to negroes from yellow fever to be no greater than that from *bilious fever* in the country, and *not half so great as from congestive intermittent.*"

Dr. DOWLER presents more explicit testimony, in the form of statistics, in regard to negro immunity. "Although non-creolized negroes are not exempt from yellow fever, yet they suffer little from it, and very rarely die. On the other hand, they are the most liable to suffer from cholera. As an example of the susceptibility of this race, take the year 1841; among 1,800 deaths from yellow fever, there were but three deaths among the blacks, two having been children, or 1 in 600, or 1 in 14,000 of the whole black population."

"The same immunity from death in this disease is enjoyed throughout the yellow fever zone by the black race; for example, in the epidemic in Charleston in 1838, the official report shows that among 538 interments of yellow fever subjects, only seven were blacks, or about 1 in 50, and these were, probably, as usual, not city creoles. This is, however, an extraordinary mortality compared with the same class in New Orleans."

"In order to ascertain the proportional number of deaths from fevers among blacks in 1853, I adopted the following plan, comprehending the six months ending November 1st: I analysed more than the one-fifth of the list of deaths by Mr. MAGINNIS, beginning with the letter A and ending at D, running through all the twelve cemeteries for the six months. The result is fourteen deaths among blacks from fevers of all kinds; two of whom, a child aged eight days, and one aged ten months, were born in New Orleans; of the residue four are mentioned under the term "child," one aged nine months, and one two years; one nine years; the birthplace of the residue, one excepted, unknown The two born in New Orleans that died, aged eight days in the one case, and ten months in the other, were doubtlessly not the children of creolized parents, many of whom are brought from more northern States and kept here for sale, not only to the citizens of the city, but to planters in the country. Among thousands of these, it would not be surprising if fourteen should die of all kinds of fever in six months, if no epidemic had existed, and if the entire race had been unsusceptible to yellow fever. In round numbers the total mortality had been 2,500—that from yellow fever about 2,000—during the period above indicated, in which fourteen blacks, many of whom were infants, died of fevers of all varieties, in a black population of 30,000. Had these deaths all been from yellow fever, they would not, so far as this worst of epidemics goes, affect the argument that while the black race is susceptible to yellow fever, if born out of the city, death is, from the disease among them, a very rare occurrence; a majority of city practitioners never, perhaps, saw a single fatal case."

This immunity of the black race is wholly incompatible with the rumor, which prevailed during the epidemic, that the disease was an "African Fever." Dr. Fenner scouts this notion and says, "I would like to know what is meant by the term. There is no part of Africa where so malignant a fever is generated as the yellow fever of America; and those who assert that this was an African fever, ought not only to point out its *distinguishing features*, but also show how it was introduced here. The truth is, the epidemic was nothing but an uncommonly malignant yellow fever, presenting all the characteristic symptoms of that disease as it is generally seen here, but in a more virulent degree."

Dr. Dowler is equally positive on this point: he remarks that "it does not appear that yellow fever prevails under an African sun; although the epidemic in New Orleans in 1853 came well nigh getting the name "African yellow fever," "African plague," it was for weeks so called!

The African is the only race to which this exemption attaches; "the Necropolis of New Orleans represents all the fundamental types, if not every variety of the human race, Caucasian, American, Asiatic, and African—all of which except the latter—become the ready victims of yellow fever, the creolized excepted. I have seen in the same day, the copper-colored American from the low lands of the Mississippi, and the Scandinavian from the icy mountains of Norway, dying of the epidemic. The Indian race is equally susceptible as the white race to yellow fever, although some writers have denied this."

The Howard Association had under its care, during the epidemic 11,088 patients; "of this number (5,845) much more than half were Irish; German, (2890) nearly a quarter; French 436; United States (716) less than one in sixteen of the whole. Hence, it appears that Ireland and Germany give 8,735; other countries 2,353."

"Omitting Spain and the United States, the yellow fever zone contributed but nineteen; the plague zone of the East, as Palestine and Greece, but seven to this formidable aggregate of 11,088."

Dr. Dowler in his "Tableau of Creole mortality in 1853 from yellow fever," adduces exceedingly interesting statistics bearing upon the disputed question as to the liability of Creoles to this form of fever. Dr. D. is very decided in his opinion upon this question; he avers that "congenital city creolism, that is the constitutional modification incidental to the being born of Creole or thoroughly creolized parents, with continuity of city residence, exempts the individual from yellow fever with nearly the same uniformity that vaccination prevents the small-pox or the varioloid. The varioloid is, as all know, modified small-pox, happening to one who has undergone vaccination, or the small-pox previously, the frequency of which is probably as great as the frequency of yellow fever among city Creoles who have never absented themselves for one or more winters in Northern climates."

In order to comprehend the full and precise import of this opinion, it is necessary to understand clearly what Dr. Dowler means by Creolism; he restricts the term in such a manner as to exclude many who have heretofore been loosely ranked as Creoles by writers on yellow fever. "In Louisiana, every native, be his parentage what it may, is a Creole. They are convertible terms."

"Although the word *Creole* in its usual acceptation means a white person, it applies to all races, as Creole negroes; it even applies to the inferior animals and things; a creole chicken, egg, or cow, is worth nearly twice as much as one from a distant State; while a creolized horse, after considerable risk, becomes better, being larger, than a creole horse. City creolization, whether native or acquired is a practical distinction in the business of New Orleans."

"The word Creole is generally used in sense too latitudinarian for precise statistical investigation. It is the resident city Creole, not the Creole who migrates every summer to New York, London or Paris, that may hope for as good health as is possible to humanity, while two or three hundred others daily fall victims around him—a definition which excludes a great many called Creoles and one often forgotten, in writing on the subject of yellow fever. Hence arises many apparent contradictions among authors who use the word in different senses."

"In former, still more than in recent times, has this fundamental distinction been overlooked. In a great majority of the works on yellow fever in the West Indies, and even in Louisiana, where Creoles are said to suffer from this disease, the true explanation is, that these persons are *Creoles of the country, not of the city;* or at most, they reside in the latter occasionally, chiefly in the winter, and are, therefore, liable to the disease, though they usually have it in a milder form than strangers, and rarely die."

Dr. DOWLER divides Creolism into the three somewhat distinct varieties of urban or city, rural, and acquired. It will be perceived from the quotation made above, that immunity from yellow fever is enjoyed most largely and indeed almost completely, according to Dr. DOWLER, only by the native city creole, with a continuity of city residence. Country creoles are not so insusceptible; "all born beyond the limits of the city are susceptible to yellow fever on coming into the city or into a village where yellow fever prevails. In 1853, yellow fever has, for the first time perhaps, prevailed to some extent in the rural districts, remote from towns, among isolated persons who had not visited them. But in almost all of these instances the disease prevailed in aggregations of people, which are virtually towns, as the plantations where the population is concentrated at one centre, often forming a village of from 100 to 500 or more persons."

Again, the mere fact of being born in the city is not in itself protective. "Thousands are thus born of uncreolized parents, who pass through the city, as immigrants, or who reside in the city in the winter only. Their return to the city might, in this way swell the number of the so called Creoles to hundreds every epidemic." A long continuous residence in the city is almost equivalent in its protective influence to native creolism, and the constitutional modification thus produced is not limited to the immediate subject of it, but is transmissible to the progeny. Creolization in a city, whether native or acquired, will exercise its protective force in any part of the yellow fever zone. On these several points, Dr. Dowler is very explicit. He says, "Creolization in the city, with or without having had yellow fever, is equal as a protection against yellow fever, to congenital or native creolism. This immunity is usually acquired in less than ten years, often in five, but to this rule very many exceptions occurred in the very extraordinary or exceptional epidemic of 1853."

"City immunity, native or acquired in similar cities, as New Orleans, Charleston, Mobile, Pensacola, Havana, Vera Cruz and other places in the present limited yellow fever zone, is probably identical and mutually protective in all such places, while nativity in cities once in the yellow fever zone in which yellow fever has not been prevalent for many years, as in Baltimore, Philadelphia, New York, Boston, Cadiz, Seville and other places affords no protection."

"City creolism both native and acquired is, to a great degree, as before remarked, hereditary or transmissible from parents to children. At least the exceptions to this law are few and fatal, results almost unknown, as may be proved by the bills of mortality though this is like many other indubitable truths boldly denied, particularly since the decline of the epidemic of 1853—the most mortal, erratic and extraordinary ever seen in New Orleans. It will have been seen what warrant the terrorists have for denying creolization."

"Setting aside the epidemic of 1853 and reasoning from what is fully proved by the past—the best exposition of the present—it will be seen what little foundation there is for the utter rejection of creolism and acclimatization, which in former years was rung and is still ringing in the public ear."

As the questions, whether Creoles enjoy immunity from yellow fever or not? whether Creoles die, and in what proportion? are exceedingly interesting, and susceptible of solution to some extent by statistics, we shall quote somewhat fully from Dr. Dowler the facts which he has with great care and labor collected, touching these points. He says, "the method which I have adopted is as fair as can be conceived, and will give in this particular a compendious view. I selected the two first weeks of August, the middle of the epidemic, as distributed among the twelve cemeteries, and among the different letters of the alphabet, counting all the persons who died of fevers of what kind soever, as the list does not distinguish the fevers—the one from the other—an arrangement the most unfortunate for the merits of the Creole question, because, in any case it will be admitted that Creoles would be more likely to die of any other fever than yellow fever. It will be provisionally assumed that all the deaths marked "fever" were from yellow fever alone. The whole number of deaths during this period among natives of New Orleans is but 21—of this number one was aged five, and two aged seven days; two aged two, one aged three and one ten months; one aged one, three aged two, one aged four, one aged five and two aged nine years. In one case the word "child," in another "infant," are used to denote the age, and in two cases the ages are omitted. Omitting the two infants and the two of unknown ages, probably infants, the average of the remaining seventeen is twenty-six months, or by omitting the two highest ages, the average age will be less than fifteen months. The list here referred to shows that these twenty-one children are all that died of fevers of every kind among all classes of population during fourteen days in the midst of the epidemic. For the sake of illustrating the argument, the least favorable to creolization, as before intimated, let it be assumed that all these died of yellow fever, though there can be little doubt that the whole number were born of unacclimated parents—parents who had not been creolized either by birth or long continuous residence in the city. Even the two aged nine years each, probably were born of parents only temporarily resident in the city during the cooler season of the year, whose summer residences are in the country; or they may have been born of parents who lived here nine years ago, as immigrants or visitors, and who had returned to New Orleans. But let it be admitted that both of these children were born of parents constantly resident for ten years—admit that these children had not been sent from the city to the country, or to the North among relatives, to school, &c., then let these two Creoles be compared with the whole number of deaths from yellow fever, and what will be the result, supposing that they did die of yellow fever and not from some other fever? The whole number of deaths during these two weeks, as taken from the official daily report of the Board of Health, is 2,702—from yellow fever alone 2,252—from diseases not named, supposed to be yellow fever 114; total deaths from yellow fever 2,369. According to this supposition, waiving, for the present, the consideration of former epidemics, one Creole in 1,184 died of yellow fever out of a creolized city population four or five times greater than the non-creolized or strangers."

"In order to vary the numerical consideration, I selected the first week in September, counting the number of deaths among persons who had been born in New Orleans. This was the more desirable, because at that advanced stage of the epidemic it might be supposed that it would reach persons long resident to a greater extent than in its exception, and, such indeed was the general opinion. Upon examining the alphabetical list as distributed among the twelve cemeteries, nine individuals proved to have been born in New Orleans; one aged two, one three, one seven, one eight years, two aged six months, one eight months, one eighteen months, and one mentioned as an "infant," giving an average age of thirty-four and a half months. This whole number is subject to all the contingencies in the first series; during these seven days the total mortality was 741, of which 560 were from yellow fever, and 33 under the head of "unknown," making 593, leaving 148 for all other diseases. This enumeration does not conflict with, but gives validity to, the explanation given of the former series."

"The St. Louis cemetery No. 1, which represents the wealthy French of the city and of the country, and those immigrating from France, presents the following mortuary tableau for six months, ending with November 1st; deaths from all fevers among individuals born in New Orleans, 6; one aged twenty months, four, respectively, 3, 10, 20 and 22 years, and one styled "infant." The explanations previously given apply here. That only six should die of fever in six months, had no epidemic prevailed, is remarkable, in a mortality of two hundred and six. Had all these six deaths been from yellow fever, it does not, in all probability, invalidate creolism in the least. Three were infants, one a girl, and two still young, who probably had not remained continuously in the city, and who, on returning, contracted the disease."

"The Protestant cemetery is the best mortuary representative of the wealthy creolized Americans, many of whom, however, could scarcely expect immunity during an epidemic, inasmuch as they leave the city annually, in the summer, and educate their children in the North. The interments from all fevers among the natives of New Orleans in this cemetery, for six months ending

November 1st, amount to eight, in a total of four hundred and thirty; one of these is a "child," one aged twenty-two months, three aged two years, one five, one fourteen, and one eighteen years. The latter two were, probably, as usual, truants, who did not stay at home much, if at all, but, having returned, died. Previous explanation will apply here; but, if they be rejected, the mortality from all fevers is, for this cemetery, a little over one for each month."

"The Cypress Grove Cemetery No. 1, is assimilated in character to that of the Protestant. In the former, the total number of deaths among natives of New Orleans from all fevers in six months ending November 1st, is four—one called a "child," one aged two, and one seven months, and one three years, out of 300 deaths; that is, one native infant died in every two months upon an average."

"The worst view of these facts does not overthrow the doctrine of immunity nor afford much aid to terrorists. The creolized dig but few graves in the swamp cemeteries, and if they were much damaged by yellow fever, these cemeteries would tell it."

It will be seen from these statistics that though the general rule of creole immunity is very satisfactorily established, there were yet some exceptions. This is especially true of the epidemic of 1853 which seems to have set at defiance all past experience on this and other points. Dr. Fenner limits his observations on this topic to the epidemic of 1853; we have already quoted his statement that "one of the most extraordinary features of this epidemic is presented in the fact tnat the natives of the city, *both white and colored*, have suffered severely, and many of them died of it. Children who were born since '47 have suffered most." Dr. Dowler admits these deaths among children born in New Orleans, but thinks that they may be explained in a way that will not contravene the opinion which he holds as to the immunity of genuine city creolism, native or acquired.

"It will, no doubt, be found upon a full examination of these fatal cases, that nearly all belong to the following classes and conditions; although born in the city, their actual residence has not been continuous, but has vibrated like a pendulum between the country and the town, between Northern schools and cities and New Orleans; or they have been born of unacclimated parents whose continuous residence has been less than ten years, often not that many months; or they have been born of parents one of whom is not acclimated; or, finally, they have been born while the parents resided temporarily in New Orleans, (constituting a large class,) and hence, called Creoles, who, subsequently having come to the city, fell victims, and hence, appear in the mortuary certificates as natives of the city."

Dr. Fenner in his chapter upon "The General Character of the Disease," expresses the opinion that the epidemic of 1853 was more malignant and unmanageable than any previous one; his own observation as far back as 1841 warrants this opinion, and it is substantiated by the testimony of the oldest practitioners of the city; the published statistics of this and other years are further proof to the same effect. The distinguishing diagnostic symptom, black vomit, occurred in more cases than usual, and the tendency to it was evinced more rapidly than in former epidemics. The symptoms of cerebral disturbance were severe to an unwonted degree, and delirium and convulsions were the precursors of death in many of the fatal cases. Suppression of urine was not noticed so frequently in dangerous cases as in former epidemics.

The tendency to hemorrhage, of which the black vomit is a conspicuous manifestation, was not limited to the stomach, but seemed to pervade the entire system. It often occurred to a dangerous and even fatal extent from the nose, gums, bowels and uterus; a case of death from hemorrhage of the scrotum is referred to. In this connection, Dr. Fenner makes some interesting remarks of a practical kind which deserve to be quoted at length. "Hemorrhage, however, was not always an unfavorable symptom, on the contrary, it often indicated the approach of a *salutary crisis*. Indeed, it is *the natural crisis of this type of fever when its course is not interrupted by art.* How often have I hailed with joy the appearance of a moderate hemorrhage from some *safe part*, as the nose, gums or uterus, in the critical stage of yellow fever, when it had been previously altogether uncertain how the case was going to terminate. At this stage of the disease, a small quantity of blood flowing spontaneously from any part except the *stomach* is most generally followed by the happiest effects, reducing any remaining febrile excitement and allaying nervous inquietude. When hemorrhage sets in, however, there's no telling how far it may go before it stops. Believing as I do that it is a *natural critical discharge in this type of fever.* I would recommend that no efforts be made to check it whilst it is *within the*

*bounds of safety*, but when it seems to threaten life, we should then resort to astringents, styptics, stimulants and tonics."

"Black vomit is nothing but a hœmorrhage from the stomach, and may exist in any degree, from a few particles of blood mixed, as it were, into a quantity of light glairy fluid, presenting the appearance of floating dark specks or flocculi, to that of a rapid and profuse discharge of *pure red blood.* Before this discharge takes place in any degree there is great engorgement of the mucous membrane lining the stomach. The hemorrhage that then occurs is the effort of nature to relieve this engorgement; consequently, if the patient dies without throwing up much black vomit, we find the mucous membrane greatly engorged, thickened, and red or livid; whereas, if the hemorrhage had been *very profuse*, we find the mucous membrane after death pale and softened. Hence we may explain a fact that has been observed by many, viz: that *recovery is more apt to take place after free discharges of black vomit* than after *very slight.* The reason is obvious: in one instance, nature effectually relieves the distressed organ by directly depleting its morbidly engorged blood-vessels; whilst in the other there is not a sufficient discharge to afford any relief."

"When a patient is threatened with black vomit the great indication is to remove that engorgement of the blood-vessels of the stomach which is its proximate cause."

"Black vomit and other hemorrhages rarely occur amongst full-blooded negroes, though I have heard of a number of cases this year, and had one myself. Mulattoes and Quadroons are almost as liable to them in yellow fever as white people, and quite a a number have died of hemorrhages this year. This difference between the black and mulatto must proceed from the mixture of races."

"Black vomit is quite common amongst white children with yellow fever, but they are far more apt to recover from it than adults. I shall say more of this in another place."

"Black vomit is by no means *restricted to yellow fever*, but is occasionally met with in other diseases, such as dysentery, cancer of the stomach, and the state of pregnancy."

The salutary influence of moderate hemorrhage upon the progress of yellow fever is not peculiar to this form of disease; all observant practitioners must have noticed a like beneficial effect from this cause in other diseases. For example, hemorrhage from the lungs in tuberculization of those organs very frequently retards the process of disorganization, and very much protracts the duration of many cases; in pneumonia, the copious expectoration of rust colored sputa materially mitigates the severity of the inflammation; chronic abdominal affections dependent upon or associated with congestion of the portal vessels are often greatly ameliorated and sometimes entirely subverted by an attack of hæmatemesis or melana; in typhoid fever, moderate hemorrhage from the bowels is not unfrequently the immediate precursor of a very decided improvement in all the symptoms; we have seen a number of cases of this fever in which convalescence promptly succeeded a moderate loss of blood in this way.

Dr. FENNER remarks upon another feature of the epidemic of 1853 which was not at all common in other epidemics, viz: a strong tendency to *relapse.* "After the customary subsidence of the fever, at the end of the third day, there would be a period of calmness lasting from twenty-four to forty-eight hours; and then the fever would kindle up again, and last for one day or more. This secondary fever was often very dangerous, though it frequently terminated very happily. The interval of convalescence after the first attack was sometimes so long, as to appear more like a *second attack* than a *relapse.* From two to four weeks have been known to elapse between two distinct attacks of the fever in the same person."

Under the head of "Special Observations," Dr. FENNER directs his attention to several special points, such as *recoveries from black vomit*, and *second attacks* of yellow fever; upon these points there exists a difference of opinion and experience among the profession of New Orleans. Dr. FENNER, while he admits that the chance of recovery is almost desperate, little better indeed than *one in a hundred*, when *well marked black vomit* is developed, yet had the good fortune to observe "quite a respectable number of recoveries" from this perilous condition in 1853. Two of these cases occurred in private practice and several were observed in the wards of the Charity Hospital.

The experience of several of the most respectable practitioners of New Orleans accords with that of Dr. FENNER, and he presents their testimony and the number of their cases. "Dr. CHAPPIN, House Surgeon of the Charity Hospital, gives it as his opinion, that *seven per cent* of the cases of black vomit in that institution recovered."

"Notwithstanding all this testimony to the fact of recovery from black vomit, one of the most prominent physicians of the city, who has practiced here near twenty years, tells me he never knew a case of *unquestionable black vomit* in yellow fever get well."

A like difference of opinion exists as to the liability of persons to second attacks of yellow fever. Dr. FENNER thinks that second attacks may occur under certain circumstances. He says, "I mean by this, persons who have had the disease in former years. In my history of the epidemic of 1847, I mentioned that several instances of this were observed, and the same has occurred this year. There can be no doubt that if a person have a plain attack of yellow fever during the prevalence of a *severe* epidemic, there will be but small probability of his ever having it again, provided he remains in the same place; yet the rule is by no means *invariable*, as I shall presently show. If one has it, however, during a *mild* epidemic, or when there are only a few sporadic cases, his subsequent exemption will not be near so great. Thus there were numerous attacks this year among persons who had had other attacks since 1847, but not many among those who had it that year, or any previous *strong* epidemic."

"I saw no case this year which had been attacked in 1847; but several who had the disease well marked in 1848, and subsequently."

"Dr. JONES informs me that he saw one case in a person who had yellow fever in 1847, and one who had it in 1839."

"Dr. KENNEDY says he never attended a case of yellow fever in a person whom he had treated before for that disease. Dr. K's., experience extends over a period of twenty years."

"Dr. LINDSAY tells me he treated this year at least a dozen cases who said they had had yellow fever before—some of them in 1847; but he cannot vouch for the accuracy of their statements. He had one case this year in a well marked attack. His last attack was mild and only lasted two days."

"Dr. MOSES M. DOWLER, who has practiced here seventeen years, says he never saw a second attack of yellow fever in a person whom he had himself attended, but a good many who said they had it the second time, and had been attended by others—he is disposed to believe that such instances have occurred."

"Dr. W. STONE, and Dr. SUNDERLAND also, say they never knew a second attack, and are disposed to doubt the testimony of those patients who told them they had the disease more than once Both of these gentlemen are of opinion that second attacks of yellow fever are as rare as second attacks of small pox or measles. Dr.

STONE even goes so far as to think that *acclimation once attained is never lost by removal from localities where yellow fever prevails.*"

"It is needless to multiply quotations upon this point, as similar contradictory testimony and opinions might be obtained throughout the profession. The thing is at last reduced to a *matter of opinion*, and cannot be definitely settled. Those who have made up their minds that a person can have yellow fever *but once*, will not be convinced to the contrary *by any facts that can be presented and vice versa.*"

Dr. FENNER devotes but a small portion of his history to the consideration of the "Treatment" of yellow fever; his reason for giving so limited a space to this important topic, as well as the results of the further trial of the "abortive treatment" of fever which has been so extravagantly lauded, for a few years past, by Southern practitioners, will appear in the following extract. "As the main object of this paper is to give a sort of historical narrative of the origin, progress, extent and mortality of the great epidemic which has so severely scourged our city, and the account is already drawn to so great a length, I deem it best not to enlarge it by a discussion of the debatable topic of *treatment*. I could not do justice to this subject, without devoting at least ten or fifteen pages to its consideration, and therefore will defer it to a future period. As many of my professional readers, however, may expect me to say something about the "*abortive treatment*" of yellow fever, so highly extolled in some of my previous writings, I must not pass it by in silence."

"In 1847, and ever since, till the present season, I have been able *promptly to cut short* nearly all the cases of yellow fever I was called to treat in this city; and there was but one year, (1851,) in which there was not a good deal of it to be treated. Several other highly intelligent physicians of this city have done the same. Our chief remedies for this purpose were large doses of quinine and opium, given at the outset of the fever. In reporting my satisfactory success with this treatment, I admitted that the disease treated although certainly *yellow fever*, was of rather a milder type, and that it remained to be seen whether the abortive treatment would be equally successful in a more malignant epidemic. The opportunity has been presented this year, and I must now make a candid statement of the results of my experience.

"When the epidemic broke out, I was one of the visiting physicians to the Charity Hospital, and soon had ample opportunities to test the abortive treatment by large doses of quinine and opium.

It did very well in many cases where there was a *fair opportunity to apply it*, but I soon discovered that it did not display that controlling influence over this fever which it had done over all the yellow fever I had met with for six years previously. I then fell upon a more moderate use of the sulphate of quinine, and finally gave preference to the *ferro-cyanuret*, in combination with blue mass and without opium or morphine. I was pleased with the results, and pursued this course to the end of the epidemic."

"I do not recollect to have treated any case of yellow fever without giving quinine in some form, and am willing to compare results with any physician in the city. I still believe it to be *one of the most*, if not *the most valuable* of all our remedies in yellow fever—in short, that it is just as valuable in this type of fever as it is in bilious remittent. Many physicians tell me they found the sulphate of quinine to fulfil their expectations this year as well as usual, whilst others report quite differently."

"When this epidemic broke out, the sulphate of quinine was prescribed boldly, by perhaps a large majority of the physicians of New Orleans; but from *the injudicious use of it*, which fell under my own observation in the wards of the Charity Hospital, I soon foresaw and predicted the *disrepute* into which the remedy would inevitably be brought, if things went on in that way. My prediction was fulfilled. From the *abuse* of the remedy in the hands of many who knew not how to prescribe judiciously, a popular prejudice rose up against it, and in the eyes of many it was brought into unmerited disgrace. But this has happened to almost every valuable remedy in the whole *materia medica*, and must continue to occur, as long as people will rashly venture to administer powerful medicines, who know little or nothing about medical science. Notwithstanding the obloquy which has been heaped upon this great and glorious remedy this year, it still maintains the high reputation it has long held amongst those physicians who have learned to prescribe it with judgment. If people expect to discover a *sovereign remedy for any special disease*—one that may be pitched into the human body without discrimination or judgment, whenever that disease is found, they are woefully mistaken. The *art of prescribing* medicines is of more importance to the community than the *real virtues* of the medicines; for the former is possessed by but few, whilst the drug stores abound in valuable remedies."

"From all I have heard and seen, I am sure that no one can boast this year of the *extraordinary* success of his treatment, whether he used quinine or not, *calomel* or not, large quantities of medicine or *none at all*. Yet I would by no means wish to convey the idea that the success of the experienced physicians of New Orleans has not been highly commendable this year, considering the virulence and malignancy of the epidemic, and the numerous difficulties in the way of bad nursing, &c., they had to contend with. For myself, I will state conscientiously that I lost less than nine per cent., of my cases in private practice, to which I was called *in good* time, and had a *reasonably fair chance to treat;* and about 16 per cent. of the whole. I doubt not that many physicians met with equal, if not superior success, and under all the circumstances, I do not think it should be considered discreditable. If the same success had been attained by all the physicians of the city, our population would not have been *decimated* as it has been by this dreadful pestilence."

As the History of Dr. FENNER and the Tableau of Dr. DOWLER were written with special reference to the Quarantine Question, our analysis of them, though already somewhat extended, would be incomplete were we to omit altogether what they have to say upon this topic. They are both decidedly opposed to Quarantine measures, as may readily be inferred from the quotations already made. Dr. FENNER says, "I am not prepared to maintain that yellow fever is *never* communicated *per se*, from person to person. On the contrary, I can readily conceive that, like cholera, typhus, and dysentery, it may sometimss display infectious or contagious properties to a limited extent. I have *never seen* an instance of the kind; but cases have been reported to me, upon such *reliable testimony*, that I could see no other way of accounting for them than *direct communication.*"

"Admitting it as true, that yellow fever is capable of regenerating itself in the bodies of the sick, and of thus extending from person to person in close proximity, let us pause a moment and examine *the value* of this fact. At all the places where yellow fever prevails in the United States, it appears at *distinct intervals* of different lengths. It breaks out and rages for a season, disap pears entirely, and after a lapse of time returns again. When it re-appears, how often can it be traced, with *reasonable probability*, to infected vessels, goods, or persons? If this can be done in a *considerable majority* of instances, it will be sufficient to make a *rule;* and the instances in which such a connection cannot be fairly traced, must be set down as *exceptions* to the rule. Now, I maintain, after careful observation of the outbreaks of yellow fever at New Orleans for the last twelve years, that no such connection could be reasonable traced in any instance. Nor has it been traced in a satisfactory manner in Mobile, Natchez, Vicksburg, or any other place in the United States, where this disease has prevailed. Hence the *great majority* of the physicians, and others who have had the best opportunity of observing, have come to the conclusion, that epidemics of yellow fever do not *usually*, if *ever*, originate from this source. This is also the uniform conclusion, with but *few exceptions*, of all those who have seen most of this disease in all parts of the world where it prevails, as any one can see who will examine the volumes of testimony that have been published on the subject."

"Again, yellow fever may be contracted in an infected district and conveyed in the person of the sufferer to a place quite remote. In such instances, which are very frequent, and doubtless familiar to thousands of persons in this region, *how often* is it communicated to those who come in contact with it? I will venture to say, *not as often as once in fifty cases.* Many would say, not once in a hundred."

"If, then, it be true that yellow fever is not communicated from one person to another by immediate presence or contact, independent of other influences, oftener than *once in fifty or a hundred exposures*, it would certainly make but a *rare exception* to a general rule; and this I believe to be about the true and real value of the now popular and warmly advocated doctrine of the *contagiousness of yellow fever.* It is equally applicable to *fomites* of all kinds."

"I have now admitted, for the sake of argument, all that is contended for by the most strenuous advocates of contagion, and what does it amount to?—that yellow fever may be conveyed from place to place, in ships, goods and persons; and even be communicated to those who come in contact, yet *this effect is so seldom produced* as to make but *a rare exception to the general rule.* Now, does this afford sufficient inducement for the establishment of *Quarantine*, involving heavy expense, and throwing serious impediments in the way of commerce and travel? *That is the question!* and I only wish that the Legislature, who have to decide and act upon it, were as fully informed as they ought to be, as to all that has been done in regard to it within the last fifty years. France and England, two of the most enlightened and rigid governments in the world, after enforcing Quarantine against yellow fever for half a century, are now abandoning it as *useless.* The reports that have been made to them by the able men appointed to investigate the subject, show most plainly that all that can be done, to either *check* or *prevent* the ravages of yellow fever, consists *in proper attention to cleanliness, ventilation and drainage.*"

A few extracts from Dr. Dowler will serve very clearly to show what his opinions are in regard to Quarantine.

"If Quarantine be not a fraud on the many for the benefit of the few, it is at least a superstition devoid of any philosophical evidence in some of its most fundamental details—as for instance, in its mystical adherence to the number *forty*, adopted in the dark ages (1485) by Councils or Boards of Health, being a monstrous compound of medical, legal and theological fancies, founded on the duration of the forty days flood; Mose's sojourn of forty days on Mount Sinai; Christ's fast of forty days in the wilderness of Judea, and the Lent fast of forty days in the Church ceremonial. Hence the Italian *quaranta*, from the Latin *quarantina*—forty days more or less—during which time persons, animals, goods, letters and ships are interdicted, confined, restrained by *coudous sonitaires*, or lazar housed, to the scandal of a fast age, which chides the tardy movements of the locomotive and steamship, and is barely satisfied with the velocity of lightning, which brands with the word *fogy* every thing devoid of rapid progression."

"Old Britain is more progressive than Young America in quarantine. The voluminous report against quarantine, especially in reference to yellow fever, by the Government Committee of Great Britain, submitted in 1852, foreshadows the opinion of the forthcoming report against quarantine even for the plague. That report will show what measures 'will supercede the necessity of those grievous interruptions to commerce and international intercommunication which quarantine, so universally imposed on account of plague, has hitherto occasioned.'"

"Contagionists have during this, as well as during all former epidemics, collected facts to prove their theory. A pedlar, from an infected district, arrives in a town—his pack is opened—he, the family, and many of the villagers die of yellow fever. Exactly the same occurrences (which are mere coincidences) take place a hundred times where there has been no pedlar—no box of goods opened—no travellers from the infected districts. In one town, a crate of crocks from New Orleans is said to have been the medium of transmitting contagion to the village; but at that very time nearly all the other towns for 500 miles around were falling under the malign influence of the epidemic. It would be most extraordinary if crates, boxes, passengers and pestilence should never happen to get together—not as causes and effects—but as coincidences necessary in the ordinary course of business. If the pestilence got into town before the arrival of a bale of goods, the former did not cause the arrival of the latter. If the man who opens the goods dies of black vomit, together with all of his family, a hundred other families take the disease without any such apparent exposure, and die in like manner. A planter fences up his grounds and secludes himself, family and slaves, and all escape; another does the same thing and all are attacked."

"Those not irrevocably wedded to contagion, might find it useful to study the events which have passed before their eyes within the last seven years."

"The late Mexican war furnishes the most complete refutation of the contagiousness of yellow fever in the absence of quarantine, so far as negative evidence can go. If the United States Government had tried to devise an experiment, on a vast scale, to ascertain whether yellow fever could be propagated by ships and armies it could not have achieved its purpose more effectually. In 1846, 1847 and 1848, this malady existed in Tampico and Vera Cruz, and was severe in New Orleans in 1847. The troops and the material of the army, leaving New Orleans for Vera Cruz, and Vera Cruz for the interior of Mexico, did not suffer themselves from yellow fever, nor spread contagion through the towns and country. In 1848, thousands of the returning soldiers passed through Vera Cruz in June, where yellow fever existed, and on reaching New Orleans in July and August, a few died out of fifteen thousand who remained in the city and its environs sometime without communicating any disease to the city by means of their goods, army materials, and selves. Thousands thus, without having been quarantined, remained in the city for a time, and quitted it for their homes, in other towns and places, without having communicated the disease to any one. After the reduction of Vera Cruz, yellow fever appeared, and many invalids and sick persons were sent to New Orleans and other places for treatment, in the transports which carried out the troops, yet they did not propagate the disease any where. Thus at least fifty thousand experiments made in Tampico, Vera Cruz and New Orleans, not to name other places, produced no proofs of personal or other kind of contagion, though in both the first named places yellow fever prevailed moderately among residents not acclimated."

"The Board of Health of New Orleans, in an official announcement, shows, that for the month beginning with the 26th of Nov. 1853, that 6,607 passengers from foreign parts, chiefly emigrants, had arrived at our wharves in forty-seven sea-going vessels, by the river route. Now, if we add the number which had previously arrived, to the number which has since arrived from sea, the aggregate will scarcely fall below 10,000, while by other routes, chiefly by the river, the emigrants, absentees and other unacclimated persons, as the steamboat population, coming to the city, in September, October, November and December, forty thousand more may be added, making fifty thousand—fifty thousand living experiments against possible contagion—fifty thousand exposures to all of the possible sources of contagion—the houses, goods, &c., of persons recently dead, including emanations from the sick and dying, during the decline of the epidemic and during the whole of this period—all proving harmless."

"Such vast, yet significant experiments quite overthrow those few cases where the opening of a box or bale of goods is followed by yellow fever,—mere coincidents not causes. There is not the least reason to think that the world, combined for the purpose, could create an epidemic yellow fever or even a single case in any city, street or house, upon the globe."

We might select from the history of Dr. Dowler many other passages of similar import, but enough has been presented to indicate the strong groundwork of his opinions upon the subject of quarantine. One more quotation from him in regard to the utter futility of some of the means adopted for the purpose of staying the march of the dreadful epidemic will close our notice of his paper.

"The practical method of attacking the *ens epidemicum* or epidemic entity, is no more satisfactory than the theoretical,—as the enfilading the streets with artillery, the combustion of tar, or the smoking of the enemy out of a place. Dr. Rush and others enumerate such examples. The plague left London, they affirm, as soon as coal was introduced into the city as fuel. Now the part of New Orleans most affected by yellow fever in 1853, was the very part most afflicted by coal smoke, namely, near the St. Mary's market, where the foundries of the city are concentrated, as Leed's, McFee's McCan's, Armstrong's etc. The burning of gunpowder, and artillery firing in the streets and public squares have sometimes been followed by the retreat of the *ens epidemicum*—just as the eating of a salt herring was followed by the recovery of a Frenchman and the death of an Englishman, by fever. The patriotic Board of Health, of which the philanthropic Mayor was chairman, had done all that was possible to stay the march and to mitigate the evils of the epidemic—scraped the streets—lustrated the gutters—provided for the sick—buried the dead, and wisely quarantined the influx of the *uninfected* immigrants,—they had done more; for they gave their personal attention to the sick and dying; in the midst of the crisis, the public mind swaying to and fro like the storm-stricken forest, they yielded to the prevailing opinion. At sun-set the epidemic was regularly, for a time, attacked with great guns. But gun-powder failed. It did worse. Sleep to the sick is the turning point of life—the first glimmer along the dark horizon of the yet dubious morning—sleep was broken—the intellect vibrating between reason and delirium, shattered by the clangor of arms and fever, raved with redoubled violence, and was sometimes quenched at once by a horrid convulsion amid the roar of cannon."

"Among the numerous plans advocated by many, was that of tar-burning. This, too, was tried. But the epidemic raged the more. Terror was supreme."

